# Influence of Enzymatic Acylation on the Stability and Antioxidant Properties of Cyanidin-3-*O*-Glucoside in Both Aqueous and Lipid Systems

**DOI:** 10.3390/molecules30092015

**Published:** 2025-04-30

**Authors:** Ziwei Ye, Mingyun Liu, Jingmei Lyu, Han Rong, Lujing Gan

**Affiliations:** School of Materials and Environment, Beijing Institute of Technology, Zhuhai 519088, China; cecilia_yee@bitzh.edu.cn (Z.Y.); my_liu@bitzh.edu.cn (M.L.); lv_jm@bitzh.edu.cn (J.L.); ronghan@bitzh.edu.cn (H.R.)

**Keywords:** cyanidin-3-*O*-glucoside, enzymatic acylation, stability, antioxidant properties

## Abstract

Cyanidin-3-*O*-glucoside (C3G) was used as a substrate for enzymatic acylation, and different compounds (methyl *n*-octanoate and methyl salicylate) were selected as acyl donors. Structural analysis (UV–Vis, FTIR, and HPLC) revealed the successful integration of methyl ester compounds into the structural units of C3G. The thermostability and photostability of acylated C3Gs, particularly those with methyl salicylate as the acyl donor, exhibited significant improvements. The molecular geometries of the different anthocyanins were optimized using computational chemistry, and energy level calculations were performed by using Density Functional Theory (DFT) to identify the antioxidant active site. Then, the antioxidant properties of C3G and acylated C3Gs (O-C3G and S-C3G) were studied in both aqueous and lipid systems. In aqueous systems, acylated C3Gs exhibited higher antioxidant properties than C3G in DPPH radical scavenging and hydroxyl radical scavenging assays, with cyanidin-3-*O*-glucoside salicyl acyl product (S-C3G) demonstrating the highest activity. However, the antioxidant properties varied in lipid systems. In lipid systems, acylated C3Gs displayed better antioxidant properties than C3G in POV and TBARS assays, with cyanidin-3-*O*-glucoside *n*-octanoate acid acyl product (O-C3G) showing better antioxidant properties compared to that in aqueous systems.

## 1. Introduction

**Figure 1:**
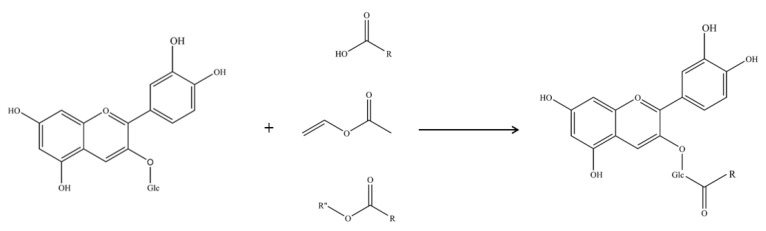
The acylation pathway of C3G with methyl esters.

Anthocyanins, polyphenolic flavonoid compounds, are kinds of natural water-soluble pigments widely found in plants. They present a variety of biological activities according to recent studies, such as having antioxidant properties and anti-aging, immunity-improving, and cardiovascular disease-preventing functions [1,2]. Compared with other flavonoid compounds, anthocyanins are more widely distributed and consumed in the human diet, and, as natural edible pigments, they have great potential for application in food and medicine [3]. However, there are many limitations since their cyclic polyhydroxy groups are easily oxidized, leading to the instability of the corresponding products. Meanwhile, the water-soluble structures also limit their application in lipid-soluble substrates. In recent years, many scientists have conducted extensive studies to address these shortcomings, and some molecular modifications have been employed to modify anthocyanins [4,5,6]. The molecular modification of anthocyanins focuses on acylation, which occurs primarily through esterification or transesterification of organic acids or esters (Figure 1), including both chemical and enzymatic methods [7,8].

Chemical modifications of anthocyanins are random, and the reaction process might have an impact on the color of anthocyanins. Compared to chemical methods, the biological enzymatic acylation reaction has better regioselectivity and has emerged as the preferred method in recent years [9]. Enzymatic acylation has high specificity (preferential action on primary alcohols of the anthocyanin glycosidic molecule) and catalytic efficiency, targeting specific groups for acylation under mild conditions [5,10,11]. Enzymatic acylation of various anthocyanins with different acyl donors has been reported. Cyanidin-3-*O*-glucoside is one of the most common anthocyanins in nature. Owing to its excellent antioxidant properties, it is frequently used as the preferred reactant in acylation [12,13]. Among the majority of studies, fatty acids are the acyl donors used for the enzymatic acylation of anthocyanins [14]. Cruz et al. [15] reported a 22–40% conversion rate of acylated anthocyanins, which was achieved by the enzymatic acylation of malvidin-3-glucoside with saturated fatty acids differing in carbon chain lengths. Several studies employing diverse aromatic acyl donor groups have also attained favorable conversion yields. In the research by Liu et al. [16], the conversion yields of *p*-coumaric acylated anthocyanins and caffeic acid anthocyanins with different acyl donors were 5.38% and 5.68%, respectively. However, few studies have compared the difference between fatty acid acyl donors and aromatic acyl donors in acylation reactions, indicating a need for additional investigations.

The properties of these modified anthocyanins are crucial for their applications. Previous studies have shown that acylated anthocyanins possess higher stability and more favorable biological activities than their unacylated forms [17,18]. To better evaluate acylated anthocyanins’ properties and bioactivities, it is crucial to assess them in diverse systems. Current evaluations focus on their processing stability, lipophilicity, and in vitro antioxidant properties. Mendoz et al. [19] found acylated anthocyanins to be more color-stable than malvidin-3-glucoside across pH levels. Zhang et al. [13] reported enzymatic acylation of C3G enhanced its thermostability, photostability, and antioxidant properties. Typically, enzymatically acylated anthocyanins’ antioxidant activity is evaluated via ABTS, DPPH, FRAP, and ORAC assays [20,21,22]. Yang et al. [12] reported a DPPH free radical scavenging activity assay and β-Carotene bleaching assay for raspberry anthocyanins and their acylated derivatives, and the antioxidant properties of raspberry anthocyanins were significantly enhanced after acylation. In recent studies, when conducting antioxidant experiments within the same system, differences have been observed. Kong et al. [23] assessed black rice anthocyanins’ antioxidant activity via DPPH, ABTS, and total antioxidant capacity assays. Acylated derivatives showed decreased ABTS activity but increased DPPH scavenging and total antioxidant capacity. Most prior studies focused on their stability and antioxidant activity in hydrophilic systems, with limited reports in lipid systems.

In this study, two kinds of methyl esters (methyl *n*-octanoate and methyl salicylate) were selected as acyl donors and enzymatically acylated to obtain two products: cyanidin-3-*O*-glucoside *n*-octanoate acid acyl product (O-C3G) and cyanidin-3-*O*-glucoside salicyl acyl product (S-C3G). The thermostability and photostability of C3G and acylated C3Gs were investigated. Theoretical calculations based on DFT analysis were performed in parallel with the evaluation of the free radical scavenging ability of C3G and C3Gs. The stability–structure relationship and the conformational relationship of the antioxidant properties of acylated anthocyanins were preliminarily investigated.

## 2. Results and Discussion

### 2.1. Enzymatic Acylation and Purification of Acylated Derivatives

**Figure 2:**
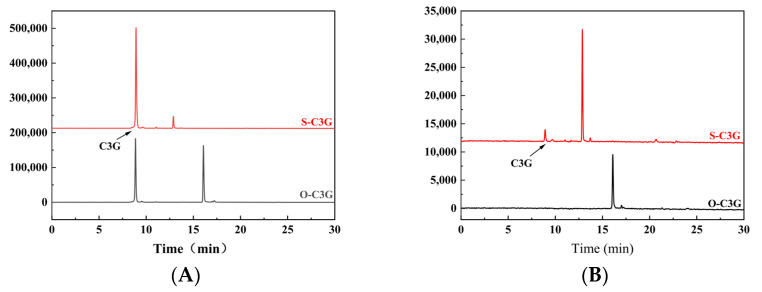
HPLC analysis of acylated C3Gs. Acylation pathway of C3G with methyl n-octanoate and methyl salicylate: (**A**); after purification of S-C3G and O-C3G: (**B**).

The reaction progress was monitored by HPLC, which revealed the formation of two new peaks (S-C3G and O-C3G) at 14.66 min and 17.77 min after the acylation of anthocyanin fractions, respectively (Figure 2). Similar results were observed by Cruz et al. [15] and Guimarães et al. [24]. The purity of S-C3G and O-C3G was 86.16% ± 0.17% and 92.79% ± 1.27% after purification, respectively. It was shown that the utilization of diverse acyl donors led to the emergence of distinct new peaks, which was attributed to the modification of the polarity of C3G by the introduced acyl donors [25]. The acylation rates of S-C3G (9.53% ± 2.20%) and O-C3G (60.04% ± 1.24%) indicated that fatty acid acyl donors (O-C3G) outperformed aromatic donors (S-C3G). The lower rates of S-C3G may be attributed to steric hindrance, where the presence of a benzene ring hinders access to the enzyme’s active site, as suggested by Marquez-Rodriguez et al. [26].

### 2.2. UV–Vis Analysis

**Figure 3:**
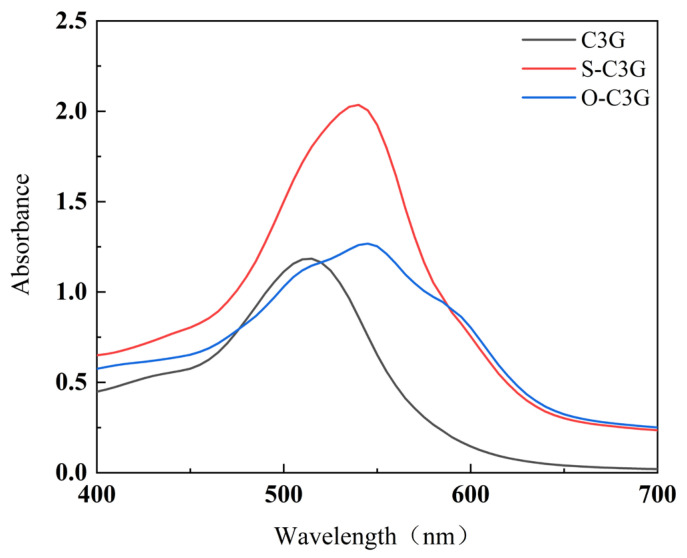
Visible light absorption spectra of C3G and acylated C3Gs (pH = 3).

UV–Vis spectroscopy revealed the impact of ester grafting on C3G’s molecular structure. Comparing the absorbance spectra of C3G, its acylated forms (Figure 3) showed slight bathochromic shifts, with λ_max_ increasing from 520 nm to 539 nm (S-C3G) and 545 nm (O-C3G). This aligns with previous studies [14,27,28], which observed similar bathochromic shifts in the acylated C3Gs. The acylation reaction not only affects the spectral properties of C3G but also influences its molar extinction coefficients. Specifically, the molar extinction coefficients for C3G, S-C3G, and O-C3G were found to be 1149.46 mL/(mol·cm), 2462.79 mL/(mol·cm), and 1549.62 mL/(mol·cm), respectively. This confirms that acylation enhances the color intensity of C3G. Notably, S-C3G displayed a more pronounced co-chromatographic effect and a higher molar extinction coefficient compared to O-C3G. This is attributed to the steric hindrance posed by the benzene ring (from aromatic acyl groups) on anthocyanins and the strengthened intramolecular co-chromatographic effects facilitated by π–π stacking interactions [29], thereby rendering S-C3G with a more intense coloration.

### 2.3. FTIR Analysis

**Figure 4:**
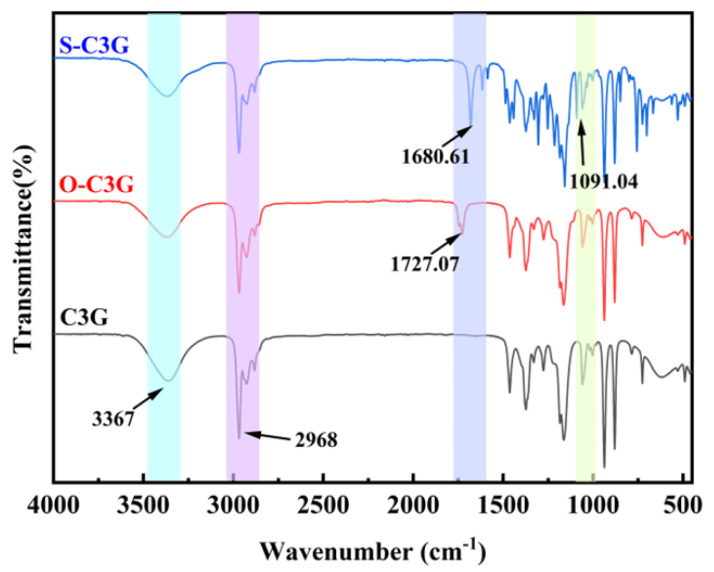
Infrared spectroscopic analysis of C3G and acylated C3Gs.

FTIR spectroscopy was employed to observe the effect of enzymatic catalysis on the functional groups of C3G (Figure 4). The strong and broad absorption band of C3G at 3367 cm^−1^ was attributed to the stretching vibration of -OH, including the hydroxyls from the aromatic skeleton and glycosyl moieties in the acylated C3Gs (S-C3G and O-C3G). The stretching vibration at 2968 cm^−1^ is caused by the stretching vibration of the methylene group -CH_2_, -CH_3_, and -CH, consistent with previous studies [30]. Compared with C3G, an absorption peak was observed within the range of 1680–1730 cm^−1^ in the spectrum of acylated C3Gs. This peak was caused by the C=O stretching vibration of the ester group, confirming the presence of carbonyl groups [31]. The C=O stretching vibration of carboxylic esters in linear structures absorbs around 1730 cm^−1^. However, esters featuring C=C-COOR or Ar-COOR (Ar represents aromatic groups) exhibit a shift to a lower wavenumber, approximately 1720 cm^−1^, due to the conjugation effects [32]. Our study confirms this by observing a distinct range of C=O stretching vibrations at 1680–1730 cm^−1^ among the acylated C3Gs. It is notable that the vibrations of S-C3G in the range of 1250–1500 cm^−1^ are different from those of C3G and O-C3G. The vibration observed at 1091.04 cm^−1^ is tentatively attributed to the vibration of phenolic hydroxyl groups [16], which is caused by the phenolic hydroxyl group present in the structure of the introduced methyl salicylate moiety.

### 2.4. Stability of C3G and Acylated C3Gs

**Figure 5:**
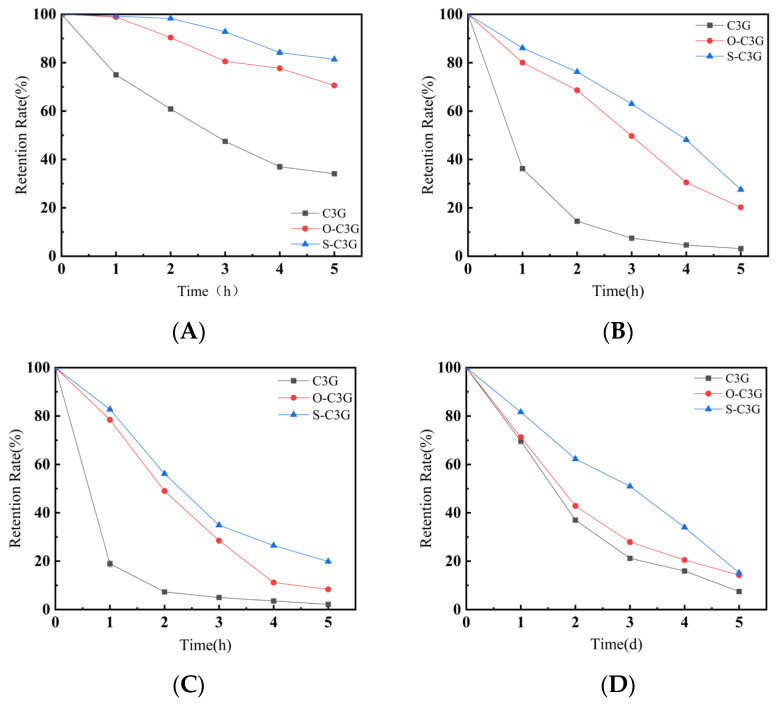
Effect of heat and light treatment on the retention of C3G and acylated C3Gs. 75 °C: (**A**); 85 °C: (**B**); 95 °C: (**C**); light treatment: (**D**). Data shown are the average of duplicate samples. Error bars on the chart represent standard deviation.

The thermostability and photostability of anthocyanins and acylated C3Gs are presented in Figure 5. In general, the retention of C3G and its acylated products decreased as the treatment time progressed. The acylated C3Gs (S-C3G and O-C3G) exhibited higher stability than their unacylated counterparts, regardless of the heating temperature and light duration, consistent with previous reports [12,22,33,34]. As shown in Figure 5A, the retention rate of C3G decreased from 100% to 34.1% after heat treatment at 75 °C for 5 h, whereas the retention rates of S-C3G and O-C3G at the 5th hour were 70.56% and 81.39%, respectively, following the order of S-C3G > O-C3G > C3G. This trend was maintained under both 85 °C (Figure 5B) and 95 °C (Figure 5C) and light treatments (Figure 5D). The thermostability of C3G and acylated C3Gs decreased with increasing temperature (Figure 5B,C). Anthocyanin, as a reducing agent, tends to lose electrons in an exothermic process that involves the benzopyran cation, hydrolysis, and ring-opening reactions [35], making them highly sensitive to temperature fluctuations and light exposure. Acylation with organic aromatic acids or fatty acids can effectively prevent anthocyanin transformation [36]. This modification allows glycosidic bonds with acyl groups to wrap around the 2-phenylbenzopyran core, forming a lamellar structure that shields the anthocyanin nucleus between organic acids [37], thereby enhancing protection against nucleophilic attack of water, degradation, and polymerization [38]. The type of acyl donor also had a significant effect on the stability. S-C3G exhibits higher stability than O-C3G (*p* < 0.05), which may be attributed to the hydrophobicity, flexibility of the glycoside, and the possibility of rotation of the B-ring relative to the polarizable and planar benzene parallel rings, which together lead to the folding of the planar aromatic acyl group on the benzene ring [39], forming a π–π conjugation effect. This results in π electron delocalization across the conjugated system rather than on the glycosidic bonds of confined anthocyanins [25,40], reducing system energy and increasing molecular and photostability. Additionally, the hydroxyl groups introduced by methyl salicylate also form a more stable internal hydrogen bond [41], and the aromatic acyl group in S-C3G absorbing light energy protects the benzopyran ring from degradation and isomerization [42].

### 2.5. Antioxidant Properties in Aqueous Solution Systems

**Figure 6:**
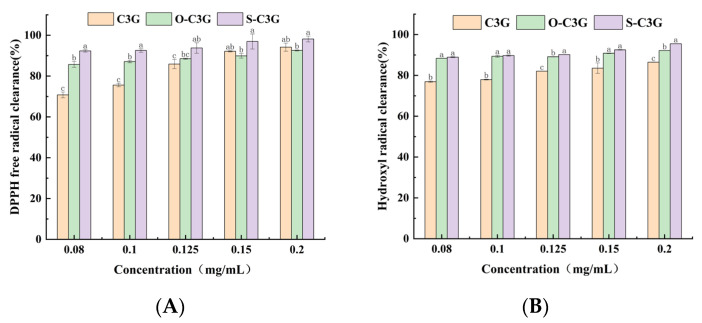
The radical scavenging ability of C3G and acylated C3Gs. DPPH free radical scavenging: (**A**), hydroxyl radical scavenging: (**B**). At the same concentration, different superscript letters represent significant differences, with a significance level of *p* < 0.05. Data shown are the average of duplicate samples. Error bars on the chart represent standard deviation.

The DPPH and hydroxyl radical scavenging ability of C3G and acylated C3Gs are shown in Figure 6. All compounds’ scavenging rates rose with increasing concentration (*p* < 0.05). The acylated C3Gs demonstrated significantly higher scavenging rates than C3G at low concentrations of 0.08 to 0.125 mg/mL (*p* < 0.05), indicating that their abilities to combine DPPH free radicals were enhanced (Figure 6A). Especially in the hydroxyl radical scavenging assay, the acylated C3Gs displayed significantly higher hydroxyl radical scavenging ability than C3G (*p* < 0.05) throughout the entire range of observed concentrations (Figure 6B). Several studies showed improved antioxidant capacity after acylation [43,44,45]. The acyl groups methyl n-octanoate and methyl salicylate have high lipophilic properties. They enwrap the C3G structure, which efficiently reduces the hydration degree of C3G and diminishes its polarity. The reduced polarity and hydration degree may limit the accessibility of free radicals and therefore enhance the antioxidant activity [12]. However, at high concentrations of 0.15 to 0.2 mg/mL, C3G and acylated C3Gs show no significant difference in DPPH radical scavenging (*p* > 0.05), a finding echoed in prior studies [14,46]. Steric hindrance might block radical scavenging sites, reducing activity at suboptimal concentrations [47]. This hindrance can both promote and inhibit scavenging, depending on factors like concentration. Notably, S-C3G excels in DPPH scavenging at all tested concentrations, likely due to its salicylic acid moiety. Despite hydroxyl group replacement, the extended conjugation from salicylic acid boosts radical scavenging, enhancing antioxidant properties [48].

### 2.6. Antioxidant Properties in Lipid Systems

#### 2.6.1. The Change of Peroxidation Value (POV)

**Figure 7:**
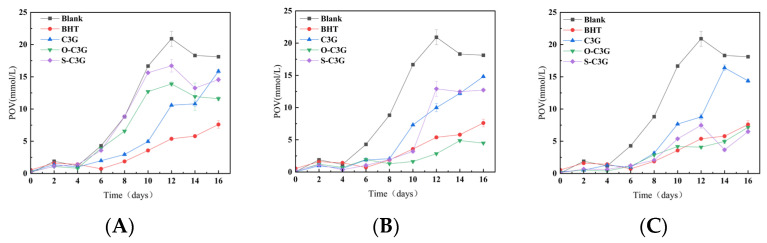
Change curves of POV in lipid system with C3G or acylated C3Gs added as antioxidants. The concentrations of C3G and acylated C3Gs were 100 ppm (**A**), 300 ppm (**B**), and 500 ppm (**C**). The concentration of BHT was 100 ppm all along. Error bars on the chart represent standard deviation.

Peroxides are generally considered to be the first products of lipid oxidation [49], and the determination of peroxide value can be used as an indicator of rancidity. As shown in Figure 7, the changes in the peroxide values of soybean oil with C3G and acylated C3Gs were observed for 16 days. All samples showed a higher inhibition ability of peroxide formation than blanks, and anthocyanins have also been shown in previous studies to inhibit lipid peroxidation [50,51].

At the low concentration of 100 ppm (Figure 7A), C3G showed greater resistance to lipid peroxidation than acylated C3G for the first 12 days. And at the end of the detected period, C3G and acylated C3Gs showed similar resistance to lipid peroxidation. At the concentration of 300 ppm (Figure 7B), acylated C3Gs consistently showed better resistance to lipid peroxidation than C3G. In particular, O-C3G showed a similar resistance to lipid peroxidation as the positive control, similar to the findings of Strugała et al. [52]. This may be attributed to the fact that acylation enhances lipophilicity, which makes O-C3G and S-C3G more effective at mitigating the diffusive effects of free radical chain reactions [53]. At the high concentration of 500 ppm (Figure 7C), as a similar result to 300 ppm, acylated C3Gs showed better resistance to lipid peroxidation than C3G. Notably, S-C3G demonstrated much stronger antioxidant properties than even the positive control after 12 days. Overall, the acylated C3Gs were more efficiently distributed in the lipid system, close to the point of peroxide production [54], exhibiting the higher inhibition ability of peroxide, whereas O-C3G generally showed better antioxidant properties compared to S-C3G, which might be due to the stronger lipophilic nature of the *n*-octanoate acid compared to the salicylate group.

#### 2.6.2. The Change of Malondialdehyde Content (TBARS)

**Figure 8:**
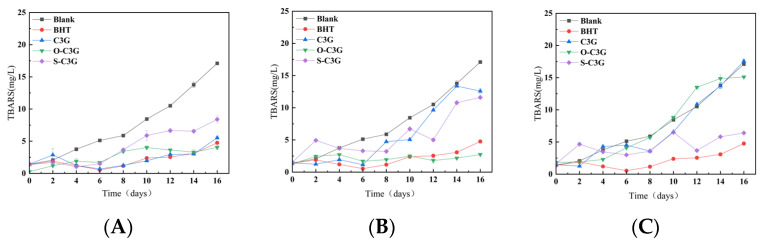
Change curves of TBARS in lipid system with C3G or acylated C3Gs added as antioxidants. The concentrations of antioxidants were 100 ppm (**A**), 300 ppm (**B**), and 500 ppm (**C**). The concentration of BHT was 100 ppm all along. Error bars on the chart represent standard deviation.

The determination of malondialdehyde by TBARS serves as a key indicator of secondary oxidation products, such as saturated aldehydes, 2-enylic aldehydes, and 2-dienylic aldehydes, produced during peroxidation of lipid. The changes of the sample’s TBARS over 16 days are shown in Figure 8, and the TBARS values of all samples also steadily increased. The inhibitory effect of C3G on TBARS varied at different concentrations, with C3G showing good inhibition at low concentrations and little inhibition at high concentrations. This may be explained by the presence of a certain amount of additives in the bulk oil. C3G may interact with it and affect the results of TBARS detection. Marteau et al. [55] have demonstrated that antioxidants with different structures together lead to different interactions, but the mechanism of reaction between C3G and antioxidants present in bulk oil still needs to be further explored. In addition, at high concentrations, C3G may decompose or polymerize [56], thus losing its antioxidant properties. Additionally, C3G probably forms aggregates in lipids due to solubility and dispersion issues [57], which may reduce its exposure to free radicals and thus reduce its antioxidant properties.

At the concentration of 100 ppm (Figure 8A), all samples showed good inhibition of TBARS compared to blanks. O-C3G and C3G showed similar inhibition to TBARS to the positive control during the detected storage. At the concentration of 300 ppm (Figure 8B), the trend was similar to that observed at 100 ppm, with O-C3G demonstrating superior capabilities even compared to BHT. According to the results from Figure 8A to Figure 8B, there was a significant reduction in TBARS formation in the samples containing acylated C3Gs (*p* < 0.05), indicating that acylated C3Gs effectively inhibited the production of secondary oxidation products. Li et al. [53] also obtained similar results, in which acylated flavonoids of bamboo leaf reduced the production of lipid hydrogen peroxide and malondialdehyde. However, at the concentration of 500 ppm (Figure 8C), all samples except S-C3G exhibited TBARS similar to blanks after 12 days. Ryu et al. [58] also found that 0.5% (*w*/*w*) freeze-dried grape skin and seed pomace extracts in minced pork more effectively reduced TBARS than 1% (*w*/*w*) extracts. During this storage, the level of inhibition of S-C3G was increased with increasing concentration. This concentration dependence was further supported by Wang et al. [59] who reported that blueberry anthocyanins had positive concentration-dependent antioxidant activity in vivo. The connection between the molecular structure of anthocyanins and the existence of concentration dependence has not yet been clearly clarified and needs to be explored.

### 2.7. Molecular Structure Optimization and Orbital Analysis

The antioxidant properties and stability of anthocyanins are closely related to their molecular configuration [60]. According to previous research [61,62,63], the structural differences of C3G, O-C3G, and S-C3G are mainly in the hydroxyl substituent at the C6″ position of the D ring (the difference of the acyl group). Therefore, in this study, cyanidin-3-*O*-glucoside, cyanidin-3-*O*-(6″-salicylic acid) glucoside, and cyanidin-3-*O*-(6″-octanoic acid) glucoside were used for computational chemical analysis. The introduction of the acyl donor causes a change in the stability of the remaining hydroxyl bonds on the C3G backbone that are not replaced, with the angle of each bond changing [41]. In an aqueous environment, the hydrophobic force changes the flexibility of the C3G backbone and promotes the rotation of the B-ring. The dihedral angles D_5-10-9-1_ of all samples (−178.346° for C3G, 177.31562° for O-C3G, and 178.38572° for S-C3G) are close to 180°, indicating that the angle formed between the A and C rings was generally minimal. In comparison, the dihedral angle D_6′-1′-2′-3′_ of S-C3G (1.54924°) was larger than O-C3G (−0.96000°). This suggests that the benzene ring of salicylic acid forms a specific angle with the anthocyanin backbone and contributes to the overall structural stability [64]. This may be due to the fact that the benzene ring (from methyl salicylate) is stacked near the A and C rings of anthocyanins by folding. The anthocyanin backbone molecules can form a certain angle (similar to the “sandwich” structure) to stabilize the structure of anthocyanin [34]. It can be indicated that the substituted anthocyanins are more stable, which is consistent with the studies of stability.

**Figure 9:**
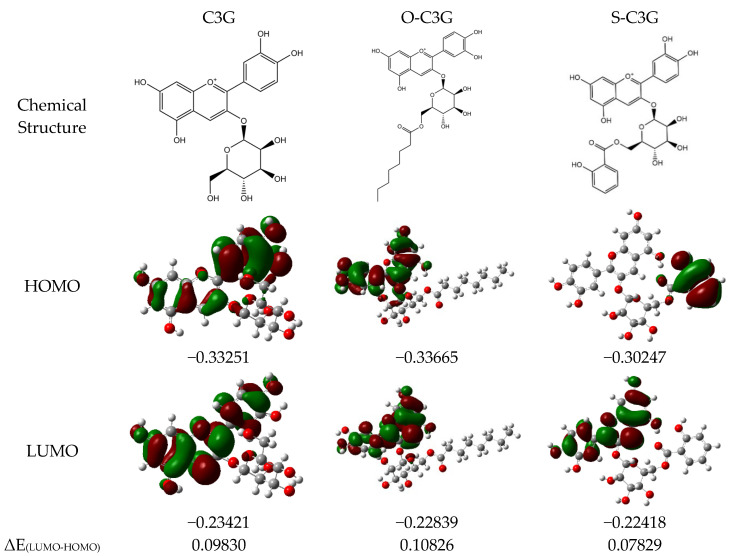
The molecular orbital composition of the HOMO and LUMO of C3G or acylated C3Gs. Red is oxygen atoms, gray is carbon atoms, and white is hydrogen atoms. The numbers indicated are the corresponding energy levels and energy level differences (a.u.). The green and red color indicate the phase of the orbital wave function, with green being negative and red being positive.

Figure 9 illustrates the distribution of the active sites involved in the reaction throughout the molecule of the acylation product. The results showed that the B ring of C3G is its main antioxidant active site, which is similar to previous studies [65,66]. In HOMO, sites with higher electron cloud density are more reactive and more susceptible to free radical attack during the reaction. Lower values of LUMO reflect greater sensitivity to nucleophilic substitution [67]. The energy level difference between HOMO and LUMO is expressed as ΔE _(LUMO-HOMO)_ in Figure 9. According to molecular orbital theory, a narrower ΔE _(LUMO-HOMO)_ means easier electron transition and stronger antioxidant activity [66]. The phenolic hydroxyl groups at C3′ and C4′ on the B ring are highly reactive. The glycosidic bond, with low electron density, does not contribute to antioxidant activity. O-C3G’s electron cloud distribution resembles C3G’s, while S-C3G’s electron cloud is on the acyl group (salicylic acid), altering the antioxidant activity site. The molecular antioxidant activity order is S-C3G > C3G > O-C3G.

### 2.8. Comparison of Antioxidant Properties in Different Systems

Overall, the antioxidant behavior of acylated C3Gs varies across different systems. Theoretical calculations were employed to determine the ranking of antioxidant capacities for C3G and acylated C3Gs under ideal conditions. Obviously, this sequence is slightly different from the order of free radical scavenging capacity observed in experiments in vitro. According to the result (Figure 6A,B), the maximum operation of capturing radicals in the DPPH and hydroxyl assay was achieved in the acylated C3Gs at a concentration of 0.08 mg/mL. Acylated C3Gs were able to achieve excellent inhibition of both POV and TBARS at a concentration of 300 ppm. Notably, S-C3G showed excellent antioxidant properties in aqueous systems (98.19% ± 1.43% for DPPH, 95.51% ± 0.03% for hydroxyl radical), while exhibiting fewer antioxidant properties than O-C3G in lipid systems. This variation can be attributed to the ability of C3G and acylated C3Gs to scavenge free radicals in different solvent systems, which is usually influenced by their polarity and solubility in the solvent [68]. Antioxidant assessments in aqueous solution show that anthocyanins have varying radical scavenging efficiencies and degradation patterns, leading to slight differences in their antioxidant properties. Yamauchi et al. [69] compared about 60 compounds and found that ORAC values are usually higher than DPPH values. This may be due to ORAC being influenced more by hydrogen atom transfer and DPPH by single electron transfer, followed by proton transfer. However, since both target phenolic hydroxyl groups, changes in the antioxidant potency of different compounds in aqueous systems show similar trends. The antioxidant function of anthocyanins in lipid systems (like soybean oil rich in unsaturated fatty acids) depends on assay metrics, antioxidant structure, and interactions between anthocyanins and lipids. Soybean oil nutrients may promote malondialdehyde–protein crosslinking, inhibiting its reactivity with TBARS [70,71]. Antioxidant additive concentrations affect lipid stability and more research on anthocyanins–antioxidants interactions is needed [72]. In our study, association colloids formed at the oil–water interface may not provide the expected inhibition [73,74]. Acylated C3Gs, with their amphiphilic structure, might enhance lipid–water interactions and alter chemical reaction rates.

## 3. Materials and Methods

### 3.1. Materials and Chemicals

Cyanidin-3-*O*-glucoside (C3G, purity: 95%) was purchased from Nanjing Plant Origin Biological Technology Co., Ltd. (Nanjing, China). Novozyme 435 (lipase B from C. antarctica immobilized on acrylic resin) was purchased from Novozymes (Copenhagen, Denmark). We purchased 2,2-diphenyl-1-picrylhydrazyl (DPPH) and Dibutyl hydroxytoluene (BHT) from Hefei BASF Biotechnology Co., Ltd. (Hefei, China). Additionally, 1,1,3,3-Tetraethoxypropane was purchased from Maclin Biochemical Technology Co., Ltd. (Shanghai, China). All other chemicals, esters, and organic solvents used were of analytical grade.

### 3.2. Preparation of Enzymatic Acylated C3Gs

The acylation of anthocyanins was performed using an enzymatic method based on the procedure described by Cruz et al. [15] with slight modification. The 2-methyl-2-butanol and methyl ester compounds (methyl *n*-octanoate and methyl salicylate) were pre-dried for at least 24 h with 4Å molecular sieves (pre-activated at 350 °C for 8 h in a muffle furnace). C3G dissolved in 2-methyl-2-butanol (1 mg/mL) was mixed with two acyl donors at a molar ratio of C3G/acyl donor of 1:150, respectively. Novozyme 435 was added at a concentration of 10 mg/mL, and the transesterification reaction was conducted at 55 °C and 240 rpm for 24 h in a refrigerated thermostatic generator (THZ-C-1, Taicang experimental equipment factory, Taicang, China). After 24 h of reaction, the Novozyme 435 lipase is filtered from the mixture and excess reaction solvent is removed by rotary evaporation. Samples were filtered by 0.22 μm nylon microporous filter after reaction. The acylated derivatives were extracted and purified with n-hexane/water solution (4:1, *v*/*v*) to remove the unreacted anthocyanin residue. Samples were collected and analyzed by high-performance liquid chromatography (HPLC).

### 3.3. Structural Characterization of C3G and Acylated C3Gs

#### 3.3.1. HPLC Analysis

The enzymatic reactions were monitored by a Shimadzu high-performance liquid chromatograph (LC-20AT, Shimadzu Corporation, Kyoto, Japan), equipped with a reversed-phase column (4.6 mm × 250 mm, 5 μm, Shim-pack GIST C18, Shimadzu Corporation, Japan), maintained at 40 °C. The detection was carried out at 520 nm. The mobile phase consisted of (A) water/formic acid (99:1, *v*/*v*) and (B) acetonitrile/formic acid (99:1, *v*/*v*). The linear gradient was applied at a flow rate of 0.8 mL/min, with the following gradient: 0.01~1 min, 10% B; 1~5 min, 10~28% B; 5~30 min, 28~95% B; 30~35 min, 95~10% B; 36~40 min, 10% B. The sample injection volume was 10 μL. The acylation rate of C3G and acylated C3Gs was calculated by the following equations:

(1) $$CY\left(\%\right)=\frac{A_{1}}{A_{1}{+A}_{0}}\times 100\%$$

where CY is the acylation of acylation rate, $A_{1}$ is the peak area of acylated C3Gs, and $A_{0}$ is peak area of C3G after acylation.

#### 3.3.2. UV–Vis Analysis

The UV–Vis absorption spectra were obtained by a UV-4802 ultraviolet/visible spectrophotometer (Shanghai Uniac Instrument Co., Ltd., Shanghai, China) with the pH adjusted to 3, and the differences between C3G and acylated C3Gs were compared. Meanwhile, the molar extinction coefficients and absorption wavelengths of C3G and acylated C3Gs were compared by the Lambert–Beer law. The molar extinctions were calculated by the following equations:

(2) A=εbc

where A is the maximum absorption value of the samples, ε is the molar extinction coefficient, b is the thickness of the cupola used in the test (cm), and c is the concentration of samples.

#### 3.3.3. FTIR Analysis

Fourier transform infrared spectra were recorded using a Nicolet^™^ iS^™^ 5 FT-IR Spectrometer (Thermo Fisher Scientific (China) Co., Ltd., Shanghai, China) to identify functional groups in C3G and acylated C3Gs within the wave number region of 4000–400 cm^−1^ at a resolution of 4 cm^−1^.

### 3.4. The Stability of C3G and Acylated C3Gs

In order to conduct research on the properties and experiments of anthocyanins, a 10% DMSO aqueous solution was utilized to dissolve C3G and acylated C3Gs (S-C3G and O-C3G) into solutions, each at a concentration of 1 mg/mL.

#### 3.4.1. Determination of Thermostability

According to the method of Yang et al. [7] with slight modification, the solutions of C3G and acylated C3Gs (S-C3G and O-C3G) were diluted to the same concentration with a pH 3 buffer solution, and then transferred to a brown vial after keeping in the dark for 30 min. The samples were placed in a water bath at different temperatures (75 °C, 85 °C, and 95 °C), and each sample was taken out for detection every hour after cooling. The absorbance values of different samples were measured by using a UV-4802 ultraviolet/visible spectrophotometer (Shanghai Uniac Instrument Co., Ltd., Shanghai, China). The retention rates were calculated by the following equations:

(3) $$R=\frac{A_{Tt}}{A_{T0}}\times 100\%$$

where *A_Tt_* is the absorbance of C3G and acylated C3Gs after the heat treatment for t hours, *A_T0_* is the absorbance of C3G and acylated C3Gs after heat treatment for 0 h.

#### 3.4.2. Determination of Photostability

C3G and acylated C3Gs (S-C3G and O-C3G) solutions were diluted to the same concentration with a pH 3 buffer. Equal volumes of each solution were placed in 15 mL transparent tubes and exposed to an LED lamp in a light-shielded environment. To avoid uneven light exposure, sample positions were randomly exchanged daily. Samples were taken out every 24 h, and their absorbance at 520 nm was measured by a UV–Vis spectrophotometer to determine the retention rate of anthocyanins. The retention rates were calculated using the following equations:

(4) $$R=\frac{A_{Pt}}{A_{P0}}\times 100\%$$

where *A_Pt_* is the absorbance of C3G and acylated C3Gs after the light processing for days, *A_P0_* is the absorbance of C3G and acylated C3Gs after the light processing for 0 days.

### 3.5. Determination of Antioxidant Activity in Different Systems

#### 3.5.1. Antioxidant Properties in Aqueous Solution Systems

We extracted a specific volume of the 1 mg/mL C3G stock solution along with C3G and acylated C3Gs and proceeded to dilute them individually to prepare experimental solutions with concentrations of 0.08, 0.10, 0.125, 0.15, and 0.2 mg/mL.

DPPH free radical scavenging activities were measured according to the method of Jeyaraj et al. [75] with slight modification. Two milliliters of DPPH (0.2 mol/L, in 95% methanol) was added to 3 mL samples of different concentrations. The mixtures were kept in the dark for 30 min at room temperature, followed by an absorbance measurement at 517 nm.

Hydroxyl radical scavenging was measured according to the method of Huang et al. [76] with a little modification. We started by combining 1 mL of each of the test solutions with 2 mL of 6 mmol/L FeSO_4_ solution and 2 mL of 6 mmol/L H_2_O_2_ solution. After thoroughly mixing the components together, we let the mixture sit in the dark for 10 min to stabilize and prevent light-induced degradation. Subsequently, we added 2 mL of a 6 mmol/L solution of salicylic acid to ethanol and mixed it thoroughly. The resulting solution was put through a reaction process in a water bath at 37 °C for 60 min. Finally, we measured the absorbance value of the reaction product at 510 nm. The radical scavenging activity was calculated using the equation below:

(5) $$The\;radical\;scavenging\;activity\left(\%\right)=\left(1-\frac{A_{Sample}-A_{Background}}{A_{Blank}}\right)\times 100\%$$

where A_Blank_ is negative control absorbance (without sample), A_Sample_ is sample absorbance, and A_Background_ is sample background absorbance (without free radical reagent)

#### 3.5.2. Antioxidant Properties in Lipid Systems

This experiment was based on the method of Gan et al. [77] and modified. Acylated C3Gs were introduced into soybean oil in varying concentrations: 100 ppm, 300 ppm, and 500 ppm. Soybean oil was used as the control, while soybean oil containing 100 ppm BHT was utilized as the positive control. After thorough mixing, the samples underwent the Schaal oven method by exposure to a digital constant temperature oven set at 55 ± 1 °C for 16 days, simulating an environment that accelerates lipid oxidation. Samples were repositioned and tested every 48 h.

Each sample (20 μL) was mixed with 3 mL of methanol/1-butanol (2:1, *v*/*v*) and 30 μL of thiocyanate/Fe^2+^ solution (freshly prepared). The thiocyanate/Fe^2+^ solution was mixed with 1 mL of 3.94 mol/L ammonium thiocyanate and 1 mL of Fe^2+^ solution, which was obtained from the supernatant of a mixture of 3 mL of 0.144 mol/L BaCl_2_ (dissolved in 0.4 mol/L HCl) and 3 mL of 0.144 mol/L FeSO_4_ (freshly prepared). After a twenty-minute interval, the absorbance was recorded at a wavelength of 510 nm. Lipid peroxides were quantified from the standard curve using H_2_O_2_.

The TBA solution was prepared using 0.1125 g of TBA and 4.5 g of trichloroacetic acid, which was dissolved in 25.421 mL of 0.25 mol/L HCl. Each sample (200 μL) was mixed with 2 mL of the TBA solution and 200 μL of distilled water. The samples were placed in a boiling water bath for 30 min, then allowed to cool to room temperature before the absorbance was measured at 532 nm. TBARS values were quantified by standard curves prepared using 1,1,3,3-tetraethoxypropane.

### 3.6. Theoretical and Computational Studies

Computational calculations were performed using the Gaussian 09W program at the DFT/B3LYP level with the basis set to 6-311G (d, p). Gaussian View program was used to analyze the atomic contribution to the highest occupied molecular orbital (HOMO) and the lowest unoccupied molecular orbital (LUMO).

### 3.7. Statistical Analysis

Analysis of variance (ANOVA) was performed to determine the significance of differences (*p* < 0.05) by SPSS software (version 19.0.0, IBM SPSS Statistics, New York, NY, USA).

## 4. Conclusions

This research investigated the acylation of cyanidin-3-*O*-glucoside using different acyl donors (methyl *n*-octanoate and methyl salicylate), catalyzed by Novozyme 435. HPLC, UV–Vis, and FTIR confirmed that C3G had successfully undergone acylation, effectively incorporating acyl groups. Compared to C3G, O-C3G and S-C3G demonstrated higher thermostability and photostability, with S-C3G showing the best stability. Acylation significantly improved the antioxidant properties of C3G, and salicylic acid even changed the antioxidant active site of C3G, which highlighted the structure–stability relationship of acylated C3Gs. In aqueous systems, S-C3G generally displayed the most excellent antioxidant properties in DPPH and hydroxyl radical assays, and the antioxidant activity of the substances showed a very strong concentration dependence. In lipid systems, O-C3G showed stronger antioxidant properties than S-C3G, indicating that O-C3G may be potentially more useful than C3G as an antioxidant in lipid systems (lipid-based foods and cosmetic formulations). C3G and acylated C3Gs at 300 ppm can achieve a great level of inhibition of POV and TBARS at the same time, which provides theoretical support for the use of acylated anthocyanins as novel food ingredients.

## Data Availability

The authors confirm that the data supporting the findings of this study are available within the article.

## Abbreviations

The following abbreviations are used in this manuscript:

C3G	Cyanidin-3-*O*-glucoside
O-C3G	Cyanidin-3-*O*-glucoside *n*-octanoate acid acyl product
S-C3G	Cyanidin-3-*O*-glucoside salicyl acyl product
acylated C3Gs	Cyanidin-3-*O*-glucoside *n*-octanoate acid acyl product and cyanidin-3-O-glucoside salicyl acyl product
